# Two new species and new records of the genus *Spinolyprops* Pic, 1917 from the Oriental Region (Coleoptera, Tenebrionidae, Lupropini) ^*^

**DOI:** 10.3897/zookeys.243.3879

**Published:** 2012-11-16

**Authors:** Wolfgang Schawaller

**Affiliations:** 1 Staatliches Museum für Naturkunde, Rosenstein 1, D-70191 Stuttgart, Germany

**Keywords:** Tenebrionidae, Lagriinae, Lupropini, *Spinolyprops*, new species, Oriental region, distribution, map, species key

## Abstract

Two new species of the genus *Spinolyprops* Pic, 1917 (Tenebrionidae, subfamily Lagriinae Latreille, 1825) are described from Thailand and China (*Spinolyprops cribricollis* **sp. n.**, *Spinolyprops thailandicus* **sp. n.**). The species characters within the genus are discussed, photographs of all seven Oriental species are added, a species key for the Oriental species is compiled, and a map with the distributional patterns is provided.

## Introduction

**Figure 1.**
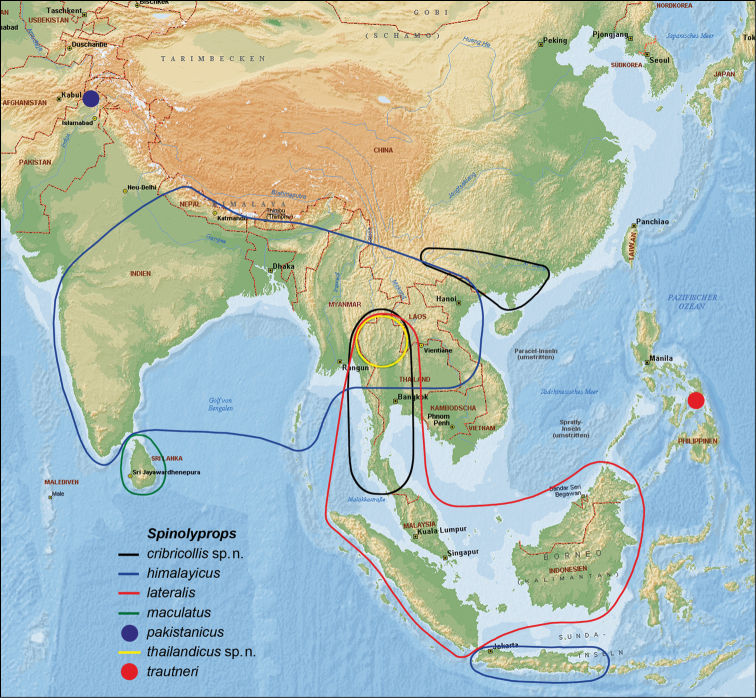
Idealized distributional patterns of the *Spinolyprops* species in the Oriental Region (modified Microsoft Encarta map).

**Figures 2–7.**
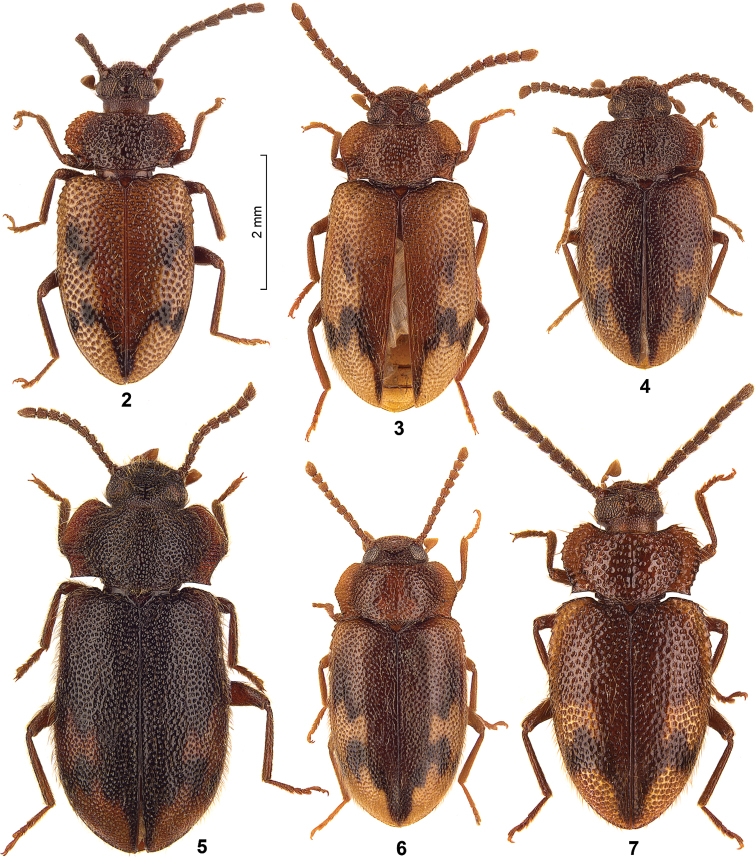
Dorsal view of *Spinolyprops* species from the Oriental Region. **2** *Spinolyprops cribricollis* sp. n., paratype Thailand CRGT **3** *Spinolyprops himalayicus*, non-type Thailand SMNS **4** *Spinolyprops himalayicus*, non-type Bali SMNS **5** *Spinolyprops himalayicus*, non-type W India SMNS **6** *Spinolyprops himalayicus*, non-type Thailand SMNS **7** *Spinolyprops lateralis*, non-type Sabah SMNS

**Figure 8–13.**
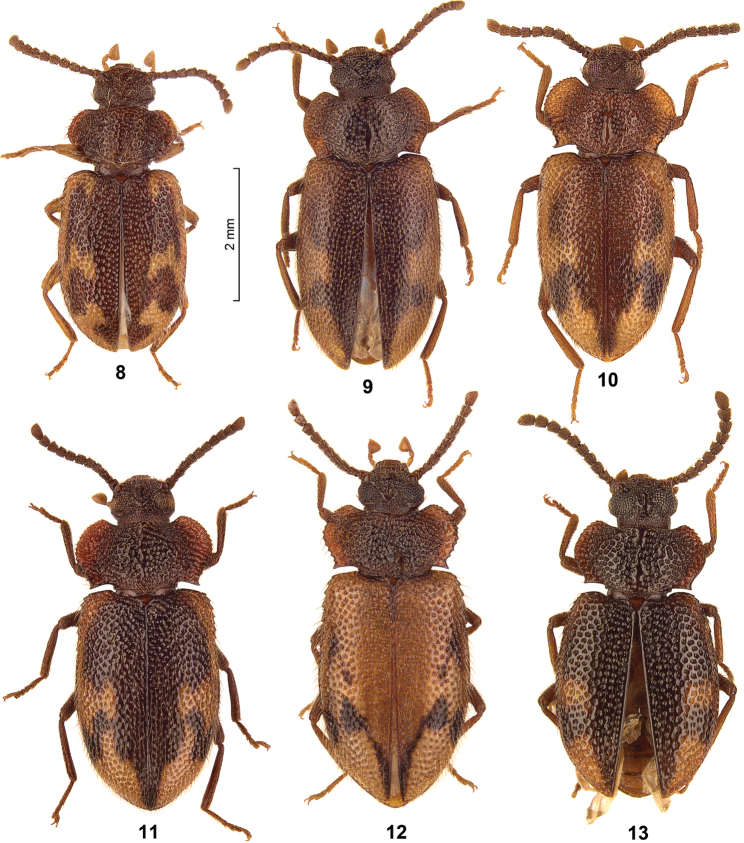
**8** *Spinolyprops maculatus*, non-type Sri Lanka SMNS **9** *Spinolyprops pakistanicus*, paratype Pakistan SMNS **10** *Spinolyprops thailandicus* sp. n., holotype Thailand SMNS **11** *Spinolyprops thailandicus* sp. n., paratype Thailand CRGT **12** *Spinolyprops thailandicus* sp. n., paratype Thailand SMNS **13** *Spinolyprops trautneri*, holotype Philippines SMNS.

The genus *Spinolyprops* Pic, 1917 (Tenebrionidae, subfamily Lagriinae Latreille, 1825, tribe Lupropini Ardoin, 1958) was based on the type species *Spinolyprops rufithorax* Pic, 1917 from Zanzibar (Pic 1917). Kulzer (1954) published the first species from the Oriental Region (Sri Lanka). Later Kaszab (1965) and Schawaller (1994, 1996) described additional Oriental species. The purpose of the present paper is the description of two further species from the Oriental region (Thailand, China), to discuss the species characters, to present for the first time photographs of all seven Oriental species (Figs 2–13), to provide a key for all Oriental species, and finally to add new faunistic data including a map with the distributional patterns (Fig. 1).

The separation of the genera *Pseudolyprops* Fairmaire, 1882, *Sphingocorse* Gebien, 1921, and *Spinolyprops* Pic, 1917 within the tribe Lupropini is still in a preliminary state and not yet based on discriminating characters. At present, the species with spine-like posterior corners of the pronotum, and with elytral colour pattern, are assigned to *Spinolyprops*. Congeners of all three genera live in Africa or in the Oriental/Papuan regions, thus zoogeographical aspects should also be considered during a future revision.

All species of this group are soil dwellers and are adapted also to extreme dry conditions (personal observations). All known species have fully developed wings and thus possess a high ability for dispersal. Specimens are usually collected by sifting litter and similar substrates, and are also attracted by light.

### Depositories

BMNHThe Natural History Museum, London (Max Barclay)


CRGTCollection Dr. Roland Grimm, Neuenbürg


HNHMHungarian Natural History Museum, Budapest (Dr. Ottó Merkl)


MHNGMuséum d’Histoire Naturelle, Genève (Dr. Giulio Cuccodoro)


MNBMuseum für Naturkunde, Berlin (Dr. Manfred Uhlig)


MSNFMuseo di Storia Naturale, Firenze (Dr. Luca Bartolozzi)


NHMBNaturhistorisches Museum, Basel (Dr. Michel Brancucci †)


NMPCNational Museum (Natural History), Prague (Dr. Jiří Hájek)


SMNKStaatliches Museum für Naturkunde, Karlsruhe (Dr. Alexander Riedel)


SMNSStaatliches Museum für Naturkunde, Stuttgart


### Species characters

**Figures 14–25.**
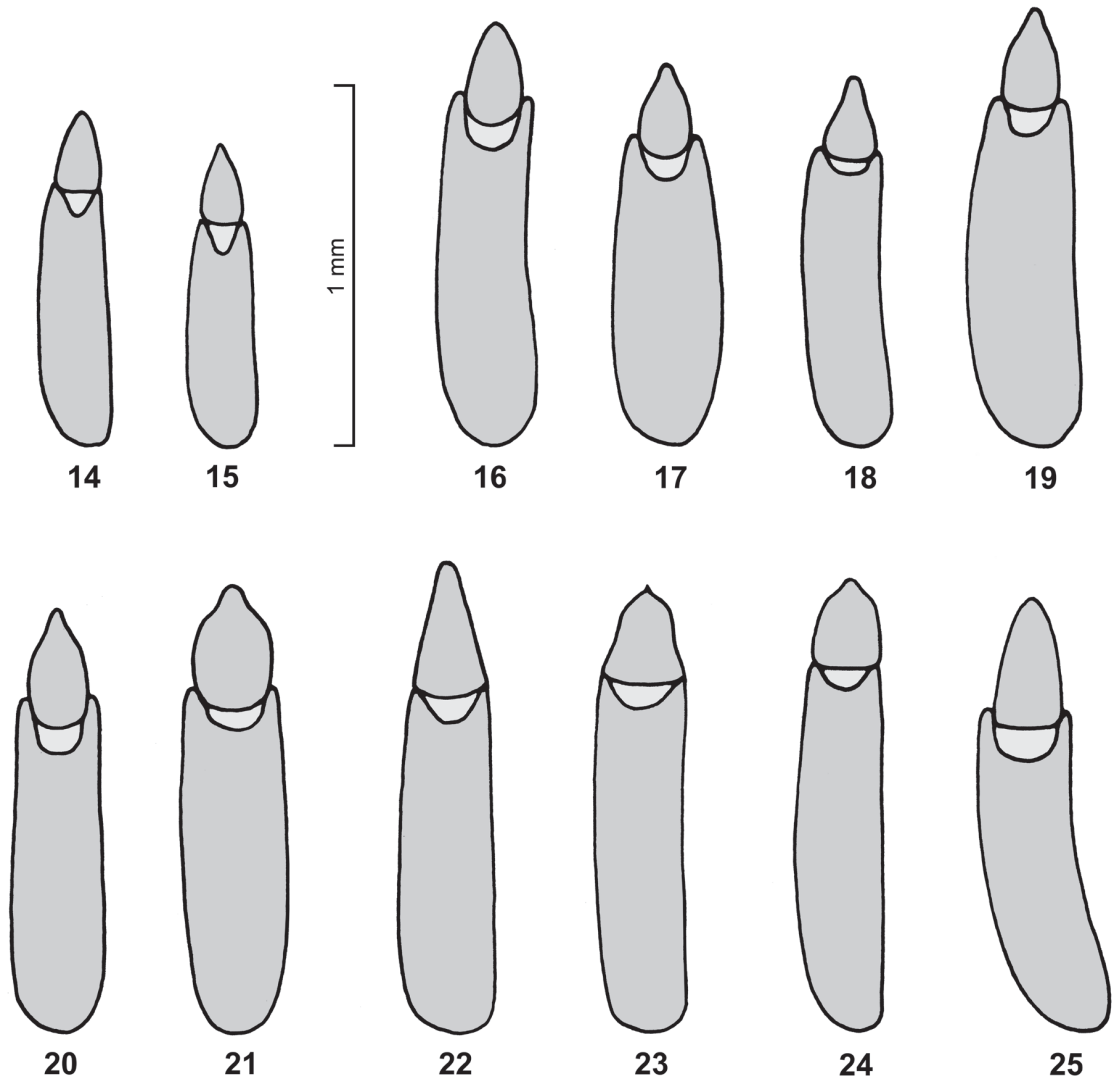
Aedeagus of *Spinolyprops* species in the Oriental Region. **14** *Spinolyprops cribricollis* sp. n., holotype Thailand/Ko Chang SMNS **15** *Spinolyprops cribricollis* sp. n., paratype Thailand/Doi Inthanon SMNS **16** *Spinolyprops himalayicus*, non-type Nepal SMNS **17** *Spinolyprops himalayicus*, non-type Bali SMNS **18** *Spinolyprops himalayicus*, non-type Thailand SMNS **19** *Spinolyprops himalayicus*, non-type W India SMNS **20** *Spinolyprops lateralis*, non-type Borneo SMNS **21** *Spinolyprops lateralis*, non-type Thailand SMNS **22** *Spinolyprops maculatus*, non-type Sri Lanka SMNS **23** *Spinolyprops pakistanicus*, paratype Pakistan SMNS **24** *Spinolyprops trautneri*, holotype Philippines SMNS **25** *Spinolyprops thailandicus* sp. n., holotype Thailand SMNS.

All species have a similar dorsal colour pattern (Figs 1–13), which is quite variable and helpful only in a low extent for species separation. On the contrary, the combination of the following characters are considered as diagnostic for species. Dorsal punctation of pronotum and elytra either fine (Figs 3–6) or rough (Figs 2, 7–13). The shape of pronotum with the lateral parts broadly (Figs 2, 7, 10–13) or narrowly (Figs 3–6) separated from disc; and with the anterior margin feebly (Figs 2–6) or deeply (Figs 10–12) excavated. Frons between eyes narrower (Figs 2, 7) or wider (Figs 3–6) than dorsal eye diameter. Antennomeres 8–10 longer than broad (Figs 2, 7) or as long as broad (Figs 3–6, 8–9). Sexual dimorphism of middle tibia present in one species (*Spinolyprops pakistanicus*), absent in all other species. Specific shape of the apicale of aedeagus, considering a certain variability (Figs 14–25). Males and females can be separated only by dissection. For separation of the species see key below (suitable only for males).

### Key to the species of *Spinolyprops* from the Oriental Region (♂)

**Table:** 

1	Frons between eyes wider than dorsal eye diameter, dorsal punctation of pronotum and elytra fine, pronotum with lateral parts narrowly separated from disc (Figs 3–6, 16–19)	*Spinolyprops himalayicus*
–	Frons between eyes narrower than eye diameter, dorsal punctation of pronotum rough, pronotum with lateral parts broadly separated from disc	2
2	Pronotum with anterior margin feebly excavated (compare figures)	3
–	Pronotum with anterior margin deeply excavated	4
3	Elytral colour pattern apically with an arrow-shaped dark element, aedeagus with long and broad triangular apicale with straight sides, antennomeres 8–10 as long as broad (Figs 8, 22)	*Spinolyprops maculatus*
–	Elytral colour pattern apically with an narrowing pointed dark element, aedeagus with short and narrow triangular apicale with rounded sides, antennomeres 8–10 longer than broad (Figs 2, 14–15)	*Spinolyprops cribricollis* sp. n.
4	Middle tibia of males on inner side with about five distinct spines (Figs 9, 23)	*Spinolyprops pakistanicus*
–	All tibiae unarmed	5
5	Separated lateral parts of pronotum extremely broad, aedeagus with broad spade-like apicale (Figs 10–12, 25)	*Spinolyprops thailandicus* sp. n.
–	Separated lateral parts of pronotum narrower, aedeagus with apicale pentagonal	6
6	Dorsal setation of pronotum and elytra short, body shape narrower (elytra 1.4× longer than broad) (Figs 13, 24)	*Spinolyprops trautneri*
–	Dorsal setation of pronotum and elytra long, body shape broader (elytra 1.3× longer than broad) (Figs 7, 20–21)	*Spinolyprops lateralis*

## The species

### Spinolyprops cribricollis sp. n.

urn:lsid:zoobank.org:act:19A94C99-40A0-4D72-9CEC-716EEA19213C

http://species-id.net/wiki/Spinolyprops_cribricollis

Figs 2 14–15

#### Type specimens.

Holotype male: S Thailand, Island Ko Chang, western side, 1999 (without detailed data), leg. A. Schulz & K. Vock, SMNS. – Paratypes: N Thailand, Chiang Mai Prov., Doi Inthanon, 1800 m, 14.V.2006, leg. R. Grimm, 4 ex. CRGT, 1 ex. SMNS. – NW Thailand, Doi Pui, 1600–1685 m, 7.–9.V.2004, leg. R. Grimm, 4 ex. CRGT. – NW Thailand, Doi Pui, 1600–1685 m, 22.–23.V.2006, leg. R. Grimm, 1 ex. SMNS. – China, Yunnan, 22 km NE Dali, NE bank of Er Hai Lake, 2010 m, 12.VI.2007, leg. M. Schülke, 1 ex. MNB, 1 ex. SMNS. – China, S Yunnan, Mengyang NR, 500 m, 12.IX.1994, leg. S. Kurbatov, 2 ex. HNHM. – China, NE Guangxi, 15 km N Longsheng, 1000 m, 15.–22.VI.1995, leg. S. Kurbatov, 1 ex. HNHM.

#### Diagnosis.

*Spinolyprops cribricollis* sp. n. shares with *Spinolyprops lateralis* the rough dorsal punctation of pronotum and elytra, the shape of the pronotum with lateral parts broadly separated from disc and bent up, the frons between eyes smaller than dorsal eye diameter, and the antennomeres 8–10 longer than broad. Both can be separated mainly by the anterior margin of the pronotum with feeble (*Spinolyprops cribricollis* sp. n.) or deep excavation (*Spinolyprops lateralis*), and by different shape of the aedeagus (in *Spinolyprops lateralis* the apicale is pentagonal, compare Figs 20–21). Additionally, *Spinolyprops lateralis* is somewhat larger in the average (5.0–6.0 mm), and the elytra are slightly more rounded. *Spinolyprops maculatus* has a similar shape of the pronotum with feebly excavated anterior margin, but the aedeagus has the apicale of the aedeagus different triangular with straight sides. *Spinolyprops trautneri* has also a different aedeagus with broad pentagonal apicale (Fig. 24). See also the species key above.

#### Description.

Body length 4.5–5.0 mm. Dorsal and ventral surfaces and all appendages brown without metallic shine, head and pronotum slightly darker, elytra bicoloured with darker and lighter parts (see Fig. 2); dorsal surface roughly punctate, punctures with long erect setae, surface between punctures shining. Head with punctation similar as on pronotum; genae distinctly swollen, clypeal suture somewhat indistinct by rough punctation, clypeus with punctation as on frons, anterior margin of clypeus straight; eyes reniform, frons between eyes smaller than dorsal eye diameter, temples impunctate; maxillary palps with large securiform terminal palpomere; shape of antennomeres see Fig. 2, antennomere 3 not elongate, terminal three antennomeres not forming club. Pronotum widest in middle, anterior and posterior margins unbordered, lateral margins unbordered but distinctly crenulate, anterior corner rounded, posterior corners acute, surface flat with irregular rough and partly confluent punctation, lateral parts broadly separated from disc and bent up; propleura with sparser and smaller punctation and shorter setation as on pronotum, prosternal process not prominent; metaventrite with punctation as on propleura. Scutellum visible, shining, without punctation. Hind wings present. Elytra elongate oval, widest in middle, lateral margin distinctly dentate in humeral region, margin completely visible from above; surface with rough punctation as on pronotum, but not confluent, punctation irregular and not arranged in rows or striae; epipleura with sparser and smaller punctation as on elytral disc, similar as on propleura. Ventrites with fine and widely separate punctation, terminal ventrite unbordered, intersegmental membranes exposed between ventrites 3–5. Legs without particular modifications, tibiae without external keels, tibial spurs short. Aedeagus with triangular apicale with acute tip (Figs 14–15). No distinct external sexual dimorphism.

#### Remarks.

I hope not to fail in assigning the (so far disjunct) Chinese specimens from Yunnan and Guangxi to the same species. Shape and punctation of the pronotum, elytral colour and shape of aedeagus are not distinctly different from the specimens from Thailand. The type locality lies in a lowland habitat (Island Ko Chang), and the paratypes from Thailand were collected in higher altitudes (Doi Pui and Doi Inthanon). Obviously, this species has a wide ecological range.

#### Etymology.

The name refers to the rough punctation of the pronotum.

### Spinolyprops himalayicus

Kaszab, 1965

http://species-id.net/wiki/Spinolyprops_himalayicus

Figs 3–6 16–19

#### Type specimens examined.

India, Darjeeling (labelled as West Bengal), Peshok, 710 ft., 19.IX.1959, leg. F. Schmid, holotype HNHM.

**New material.** Nepal, Gorkha Distr., Arughat Bazar, 600 m, 26.VII.1983, leg. J. Martens & W. Schawaller, 1 ex. SMNS. – Nepal, Surkhet Distr., Bheri Khola Bridge, 500 m, 24.–25.V.1998, leg. W. Schawaller, 1 ex. SMNS. – Nepal, Chitwan NP, Rampur, V.2005, leg. D. Ahrens, 1 ex. SMNS. – N India, Darjeeling, Sukna, 180 m, 21.–23.V.1980, leg. G. Topál, 1 ex. HNHM. – N India, Uttar Pradesh, Mussorie, 1300 m, 10.VII.1989, leg. A. Riedel, 1 ex. SMNS. – NE India, Meghalaya, 1 km E Tura, 500–600 m, 13.–18.V.2002, leg. M. Trýzna & P. Benda, 1 ex. SMNS. – W India, Maharasthra State, 70 km S Pune, Wai, 3.–6.X.2005, leg. F. & L. Kantner, 1 ex. SMNS. – W India, Maharasthra State, 40 km W Pune, Mulshi, 7.–11.X.2005, leg. F. & L. Kantner, 1 ex. SMNS. – S India, Anamalai Hills, Cinchona, 3500 ft., 1959, no collector labelled, 9 ex. NHMB (Frey collection, det Kulzer). – Bhutan, Samchi, 300 m, 7.–11.V.1972, Basel Expedition, 2 ex. HNHM. – Andaman Islands, Havelock Island, village no. 7, 22.IV.–14.V.1998, leg. K. & S. Majer, 2 ex. NHMB, 1 ex. SMNS. – N Thailand, Chiang Mai, Doi Pui, 1500 m, 19.XII.1988, leg. K. Geigenmüller & J. Trautner, 1 ex. SMNS. – N Thailand, Chiang Dao, 9.I.1989, leg. K. Geigenmüller & J. Trautner, 1 ex. SMNS. – N Thailand, Chiang Dao, 70 km N Chiang Mai, 26.–28.IV.2003, leg. O. Šafránek, 2 ex. SMNS. – N Thailand, Chiang Mai Prov., Ban San Pakia, 1700 m, 25.IV.–7.V.1996, leg. S. Bilý, 7 ex. NHMB, 1 ex. SMNS. – N Thailand, Nan, 22.–24.V.1999, leg. R. Grimm, 16 ex. CRGT, 1 ex. SMNS. – N Thailand, Nan, 2.–4.V.2003, leg. R. Grimm, 2 ex. CRGT. – NW Thailand, Doi Pui, 1600–1685 m, 15.–16.IV.2004, leg. R. Grimm, 1 ex. CRGT. – N Thailand, Chiang Mai, Doi Pui, 1600 m, 15.–16.IV.2004, leg. W. Schawaller, 2 ex. SMNS. – NW Thailand, Chiang Dao, 700–800 m, 4.V.2004, leg. R. Grimm, 3 ex. CRGT. – NW Thailand, Mae Hong Son, 5.V.2004, leg. R. Grimm, 2 ex. CRGT. – NW Thailand, 5 km E Pai, 700 m, 19.IV.2004, leg. W. Schawaller, 1 ex. SMNS. – NW Thailand, Soppong, 700 m, 23.IV.2004, leg. W. Schawaller, 9 ex. SMNS. – NW Thailand, Soppong (Pangmapa), 17.–18.V.2006, leg. R. Grimm, 5 ex. CRGT. – Thailand, Chumphon Prov., Pha To, 27.III.–14.IV.1996, leg. K. Majer, 3 ex. NHMB. – Thailand, Thanon Thong Chai, Palong, 750 m, 26.–28.V.1991, leg. V. Kubán, 3 ex. NHMB. – Thailand, Prachin Buri Prov., Sakaerat Ecology Research Institute, 4.VI.2001, leg. E. Horváth & G. Sziráki, 5 ex. HNHM. – Burma, N Shan State, Namhsan, 1500–1900 m, 18.–28.II.1996, leg. S. Kasantsev, 1 ex. NHMB. – C Laos, Khammouan Prov., Ban Khoun Ngeun, 200 m, 19.–31.V.2001, leg. L. Dembický, 5 ex. SMNS. – C Laos, Khammouan Prov., Ban Khoun Ngeun, 17.V.–6.VI.2007, leg. M. Štrba, 1 ex. SMNS. – CE Laos, Boli Kham Xai Prov., 8 km NE Ban Nape, 600 m, 1.–18.V.2001, leg. L. Dembický, 6 ex. SMNS. – Laos, Champassak Prov., Bolavens Plateau, 3 km SE Ban Lak, 1070 m, 9.V.2010, leg. J. Hájek, 1 ex. NMPC. – Laos, Champassak Prov., Ban Nong Luang, 12 km S Paksong, 800 m, 6.IV.1998, leg. O. Merkl & G. Csorba, 1 ex. HNHM. – Laos, Phongsaly Prov., Phongsaly, 1500 m, 28.V.–20.VI.2003, leg. M. Brancucci, 2 ex. NHMB. – Laos, Phongsaly Prov., Phongsaly, 1500 m, 6.–17.V.2004, leg. M. Brancucci, 1 ex. SMNS. – Vietnam, Daklak Prov., Buon Ma Thuot, Dak Linn, 500 m, 28.–29.IV.1986, leg. S. Golovatch & L. Medvedev, 7 ex. SMNS. – Vietnam, Bac Kan Prov., Ba Be NP, 350 m, 3.–8.VI.2011, leg. L. Bartolozzi et al., 3 ex. MSNF. – Java, Batavia (now Jakarta), III.1921, no further data, 1 ex. HNHM. – C Bali, Bedugul, Tamlingan, 1210 m, 6.XI.2007, leg. A. Riedel, 10 ex. SMNK, 4 ex. SMNS.

#### Distribution.

India (type locality Peshok/Darjeeling), Nepal, Bhutan, N Thailand, Vietnam (Schawaller 1996); Andaman Islands, Burma, Laos, Java, Bali (new records).

### Spinolyprops lateralis

Pic, 1917

http://species-id.net/wiki/Spinolyprops_lateralis

Figs 7 20–21

Spinolyprops rufithorax var. *lateralis* Pic, 1917

#### New material.

NE Sumatra, Tebing-Tinggi, 1 ex. NHMB (Frey collection). – E Sumatra, Lampung, Bawang, Pedada Bay, Gn. Tanggang, 660 m, 9.VIII.2006, leg. A. Riedel, 1 ex. SMNS. – Borneo, Brunei, Temburong Distr., ridge NE Kuala Belalong, 300 m, X.1992, leg. J. H. Martin, 1 ex. BMNH. – Borneo, Sabah, Crocker Range, Tenom, Kalang waterfall, 17.VI.1998, leg. J. Kodada & F. Čiampor, 5 ex. SMNS. – Borneo, Sabah, Sapulut, Batu Pungull, 24.–26.VI.1998, leg. J. Kodada & F. Čiampor, 1 ex. SMNS. – Borneo, Sabah, Poring, 650 m, 15.V.2005, leg. R. Grimm, 1 ex. CRGT. – Borneo, Sarawak, Gunung Santubong, 10–200 m, 4.–8.IV.2009, leg. R. Grimm, 1 ex. CRGT. – Borneo, Sarawak, Gunung Santubong, 30–200 m, 30.XI.–5.XII.2010, leg. R. Grimm, 4 ex. CRGT. – Borneo, Sarawak, Gunung Gading NP, 100–300 m, 31.III.–4.IV.2009, leg. R. Grimm, 1 ex. CRGT. – Borneo, Sarawak, Gunung Gading NP, 50–200 m, 8.–10.XII.2010, leg. R. Grimm, 2 ex. CRGT. – NW Thailand, Mae Hong Son Prov., 32 km NNE Mae Hong Son, 5.V.2004, leg. R. Grimm, 2 ex. CRGT. – S Thailand, Khao Lak NP, Thone Chong Fa Waterfall, 100–300 m, 6.–15.I.1998, leg. A. Schulz & K. Vock, 3 ex. SMNS, 1 ex. MNB. – W Malaysia, Perak, 25 km NE Ipoh, Banjaran Titi Wangsa Mts., Mt. Korbu, 1200 m, 6.–12.V.2001, leg. P. Čechovský, 1 ex. SMNS.

#### Distribution.

Sumatra (type locality); Borneo, Thailand, W Malaysia (new records).

### Spinolyprops maculatus

Kulzer, 1954

http://species-id.net/wiki/Spinolyprops_maculatus

Figs 8 22

#### Type specimens examined.

Sri Lanka, Colombo, III.1953, leg. G. Frey, holotype and 1 paratype NHMB (Frey collection), 2 paratypes HNHM.

#### New material.

Sri Lanka, Uva, Diyaluma Falls, 400 m, 23.I.1970, leg. C. Besuchet, I. Löbl & R. Mussard, 3 ex. MHNG, 1 ex. SMNS. – Sri Lanka, Uva, Monaragala, 300 m, 13.II.1970, leg. C. Besuchet, I. Löbl & R. Mussard, 1 ex. HNHM. – Sri Lanka, Uva, S Wellawaya, 300 m, 25.I.1970, leg. C. Besuchet, I. Löbl & R. Mussard, 1ex. HNHM. – Sri Lanka, Periyapullumalai, 11.II.1970, leg. C. Besuchet, I. Löbl & R. Mussard, 1 ex. HNHM. – Sri Lanka, Kandy, 18.III.1973, leg. G. Zimmermann, 1 ex. SMNS. – S Burma (labelled as Tenasserim), no additional data, 1 ex. HNHM (det. Kaszab).

#### Remarks.

The specimen from Tenasserim was already published by Kaszab (1965). This specimen clearly belongs to *Spinolyprops maculatus* and shares with the specimens from Sri Lanka the elytral colour pattern apically with an arrow-shaped dark element (Fig. 8). It is the only specimen of *Spinolyprops maculatus* out of Sri Lanka, so probably it was mislabelled (and is not mapped herein).

#### Distribution.

Sri Lanka (type locality), ? S Burma (Kaszab 1965).

### Spinolyprops pakistanicus

Schawaller, 1996

http://species-id.net/wiki/Spinolyprops_pakistanicus

Figs 9 23

#### Type specimens examined.

Pakistan, Hazara, Malkandi, 1500 m, 3.VI.1983, leg. C. Besuchet & I. Löbl, 2 paratypes SMNS. – Pakistan, Swat, Madyan, 1400 m, 16.V.1983, leg. C. Besuchet & I. Löbl, 1 paratype SMNS.

#### Distribution.

Northern Pakistan in Hazara and Swat.

### Spinolyprops thailandicus sp. n.

urn:lsid:zoobank.org:act:E51CF1A3-852E-4A33-8196-46C799657133

http://species-id.net/wiki/Spinolyprops_thailandicus

Figs 10–12 25

#### Type specimens.

Holotype male: N Thailand, Chiang Mai, Doi Pui, 1600 m, 15.–16.IV.2004, leg. W. Schawaller, SMNS. – Paratypes: same data as holotype, 3 ex. SMNS. – N Thailand, Chiang Mai, Doi Pui, 1600-1685 m, 23.IV.–12.V.2003, leg. R. Grimm, 4 ex. CRGT. – N Thailand, Chiang Mai, Doi Pui, 1600-1685 m, 7.–9.V.2004, leg. R. Grimm, 6 ex. CRGT. – N Thailand, Chiang Mai, Doi Pui, 1600-1685 m, 12.–13.V.2006, leg. R. Grimm, 7 ex. CRGT, 4 ex. SMNS. – N Thailand, Chiang Mai, Doi Pui, 1600-1685 m, 22.–23.V.2006, leg. R. Grimm, 6 ex. CRGT, 3 ex. HNHM.

#### Diagnosis.

*Spinolyprops thailandicus* sp. n. is characterized by the shape of the pronotum with deeply excavated anterior margin and with the lateral parts broadly separated from disc and bent up, in combination with rough dorsal punctation of pronotum and elytra and the frons between eyes smaller than dorsal eye diameter. The aedeagus of *Spinolyprops thailandicus* sp. n. is similar as in *Spinolyprops cribricollis* sp. n.(compare Figs 14–15), but in this species the lateral parts of the pronotum are not so widely separated from the disc as in *Spinolyprops thailandicus* sp. n., and the anterior margin of the pronotum is only feebly excavated. See also species key above.

#### Description.

Body length 4.3–5.3 mm. Dorsal and ventral surfaces and all appendages brown without metallic shine, elytra bicoloured with darker and lighter parts in different variation (Figs 10–12); dorsal surface roughly punctate, punctures with long erect setae, surface between punctures shining. Head with punctation similar as on pronotum; genae distinctly swollen, clypeal suture somewhat indistinct by rough punctation, clypeus with punctation as on frons, anterior margin of clypeus straight; eyes reniform, frons between eyes as broad as dorsal eye diameter, temples impunctate; maxillary palps with large securiform terminal palpomere; shape of antennomeres see Figs 10–12, antennomere 3 not elongate, terminal three antennomeres not forming club. Pronotum widest slightly before middle, anterior and posterior margins unbordered, lateral margins unbordered but distinctly crenulate, anterior corner rounded, posterior corners acute, surface flat with irregular rough and partly confluent punctation, lateral parts broadly separated from disc and bent up; propleura with sparser and smaller punctation and shorter setation as on pronotum, prosternal process slightly prominent; metaventrite with punctation as on propleura. Scutellum visible, shining, without punctation. Hind wings present. Elytra elongate oval, widest in middle, lateral margin distinctly dentate in humeral region, margin completely visible from above; surface with rough punctation as on pronotum, but not confluent, punctation irregular and not arranged in rows or striae; epipleura with sparser and smaller punctation as on elytral disc, similar as on propleura. Ventrites with fine and widely separate punctation, terminal ventrite unbordered, intersegmental membranes exposed between ventrites 3–5. Legs without particular modifications, tibiae without external keels, tibial spurs short. Aedeagus with broad spade-like apicale with rounded tip (Fig. 25). No distinct external sexual dimorphism.

#### Etymology.

Named after the type locality in Thailand.

### Spinolyprops trautneri

Schawaller, 1994

http://species-id.net/wiki/Spinolyprops_trautneri

Figs 13 24

#### Type specimen examined.

Philippines, Leyte, Lake Danao, 500 m, 19.II.–18.III.1991, leg. K. Geigenmüller, W. Schawaller & J. Trautner, male holotype SMNS.

**Distribution.** Philippines (type locality Leyte Island).

## Supplementary Material

XML Treatment for Spinolyprops cribricollis

XML Treatment for Spinolyprops himalayicus

XML Treatment for Spinolyprops lateralis

XML Treatment for Spinolyprops maculatus

XML Treatment for Spinolyprops pakistanicus

XML Treatment for Spinolyprops thailandicus

XML Treatment for Spinolyprops trautneri
